# Reconstruction of the rotation center of the hip after oblong cups in revision total hip arthroplasty

**DOI:** 10.1007/s10195-012-0217-8

**Published:** 2012-11-17

**Authors:** Eduardo García-Rey, Ricardo Fernández-Fernández, David Durán, Rosario Madero

**Affiliations:** 1 Department Orthopaedics-IDi Paz, Hospital La Paz, P° Castellana 261, 28046 Madrid, Spain; 2 Department Biostatistics-IDi Paz, Hospital La Paz, Madrid, Spain

**Keywords:** Revision hip arthroplasty, Cementless cup, Reconstruction, Outcome

## Abstract

**Background:**

The preoperative bone defect and the reconstruction of the center of rotation of the hip are critical in acetabular revision surgery. Uncemented oblong cups are employed in order to manage these issues. We analyzed the clinical results and rates of revision of two different uncemented oblong cups, the reconstruction of the center of rotation of the hip, as well as the rate of radiological loosening and possible risk factors.

**Materials and methods:**

Forty-five patients (46 hips) underwent acetabular revision surgery using two different uncemented oblong cups. We assessed the clinical results and the survival rate for revision and aseptic loosening. Intraoperative bone loss was classified according to Paprosky, and acetabular reconstruction was assessed according to Ranawat. The mean follow-up was 7.2 years (range 4–11 years).

**Results:**

There were four re-revisions (three due to aseptic loosening); the survival rate for re-revision due to aseptic loosening was 60.1 % at seven years. The mean distance between the center of the femoral head prosthesis and the approximate center of the femoral head improved from 21.5 to 10.2 mm. Thirteen cups showed radiological loosening; the survival rate for radiological loosening at seven years was 40.54 %. A smaller postoperative horizontal distance was correlated with cup loosening.

**Conclusions:**

Although optimal acetabular reconstruction can be achieved by using oblong uncemented cups in revision hip surgery, the clinical and radiological results are not encouraging. Excessive medialization of the cup may increase the rate of loosening.

## Introduction

Uncemented hemispherical acetabular cups are a common option in revision hip surgery [10, 15, 35, 37], although they have shown some limitations when major acetabular bone loss is present [12]. Different studies report the need to restore the acetabular anatomy and the anatomical center of rotation of the hip in order to obtain stable fixation of the prosthetic components, especially in revision surgery in cases with deficient bone stock [21, 40]. Acetabular bone reconstruction tries to maximize the contact between host bone and the porous-coated implant and to normalize the center of rotation of the hip, so alternative uncemented acetabular reconstruction options such as oblong cups are used. These implants attempt to augment acetabular bone contact in the critical superior zone without compromising either column [9, 22], and to relocate the anatomic center of rotation of the hip so as to avoid an excessively superior placement of the cup on the pelvis. A few recent reports have shown good intermediate results [18, 23, 36].

We hypothesized that a good anatomical reconstruction of the acetabulum would provide stable bone fixation with these types of implants in revision hip surgery. The purpose of the study described in this paper was to: (1) analyze the rate of complications, the clinical results, and the probability that re-revision surgery was not needed when two different oblong cups were employed in revision total hip arthroplasty (THA) at mid-term follow-up; (2) to evaluate the acetabular reconstruction according to the radiological parameters on the preoperative and postoperative radiographs; and (3) to assess the radiological results of these implants during follow-up, the survival rate for radiological loosening, and the possible risk factors for loosening, such as preoperative bone loss, postoperative cup position, and reconstruction of the center of rotation of the hip, using a Cox model.

## Materials and methods

Forty-five consecutive patients (46 hips) underwent acetabular revision surgery for aseptic loosening and received an oblong cup at our Institution between March 2000 and June 2007. There were 28 women and 17 men with a mean age of 71.6 years (range 30–91). The minimum follow-up period for unrevised hips was four years. The mean follow-up for all hips until revision or the last follow-up evaluation was 7.2 years (range 1–11). The mean time between primary surgery and revision was 11.8 years (range 2–22). This was the first revision surgery in all cases. Intraoperative acetabular bone loss was classified according to Paprosky et al. [31] as: type 2B, 17 hips; 2C, 12 hips; type 3A, 15 hips; type 3B, two hips. The patients’ demographic and removed cup data are shown in Table 1. No patients were lost to follow-up. Oral and written informed consent was obtained from all patients, and they were informed preoperatively that they might receive an oblong cup. This study conforms to the Declaration of Helsinki, and the institutional review board of our institution approved it.

**Table 1:** Patients and operative data

Case no.	Age of patient (years)	Gender	Primary diagnosis	Primary cup	Years to revision	Bone defect [13]	Implant	Size	Allograft
1	74	Male	OA	Omnifit	15	2B	Bofor	56-6	Yes
2	75	Female	OA	Charnley	22	2B	Bofor	56-6	Yes
3	58	Female	Dysplasia	Balgrist	10	3A	Bofor	52-6	Yes
4	75	Female	OA	Charnley	22	3A	Bofor	60-12	Yes
5	75	Male	OA	Omnifit	8	3A	Bofor	60-12	Yes
6	74	Male	OA	Omnifit	8	2C	Bofor	60-6	Yes
7	78	Male	OA	Elite	6	2B	Bofor	56-6	Yes
8	80	Male	Postraumatic	Charnley	2	2B	Bofor	60-6	No
9	75	Female	OA	Omnifit	12	3B	Bofor	56-6	Yes
10	48	Male	Postraumatic	Omnifit	4	2B	Bofor	56-6	Yes
11	82	Female	OA	Balgrist	16	3A	Bofor	60-12	Yes
12	72	Female	OA	PCA	15	3A	Bofor	48-10	Yes
13	72	Female	OA	Omnifit	8	2C	Bofor	48-10	Yes
14	86	Male	OA	Omnifit	10	2C	Bofor	60-6	Yes
15	71	Female	OA	Profile	13	2B	Bofor	60-6	Yes
16	68	Female	Reumatoid arthritis	Profile	3	3A	Bofor	65-6	Yes
17	78	Female	OA	Omnifit	9	2C	Bofor	60-12	Yes
18	69	Male	OA	RM	13	2B	Bofor	56-12	Yes
19	72	Male	OA	Charnley	30	3A	Bofor	56-6	Yes
20	72	Female	OA	Omnifit	11	2B	Bofor	52-6	Yes
21	70	Male	OA	Balgrist	4	2B	Bofor	60-12	Yes
22	85	Female	OA	Elite	6	2B	Bofor	56-6	Yes
23	75	Male	OA	Müller	20	3A	Bofor	65-6	Yes
24	50	Female	Dysplasia	Omnifit	11	2B	Bofor	52-6	Yes
25	80	Female	OA	Omnifit	12	2C	Bofor	48-5	Yes
26	69	Female	Postraumatic	Müller	9	2B	Bofor	60-6	No
27	80	Female	OA	Omnifit	16	2B	Bofor	52-6	No
28	82	Female	OA	Charnley	19	3A	LOR	64-12	Yes
29	74	Female	OA	Balgrist	6	3A	LOR	52-6	Yes
30	68	Female	OA	Plasmacup	7	2B	LOR	56-12	No
31	76	Female	OA	PCA	12	3A	LOR	56-2	Yes
32	69	Female	OA	PCA	16	2C	LOR	56-62	Yes
33	32	Male	OA	PCA	9	2C	LOR	52-6	Yes
34	83	Male	OA	Alloclassic	10	2C	LOR	60-12	Yes
35	82	Female	OA	Charnley	11	2B	LOR	56-6	Yes
36	81	Female	OA	Charnley	10	3A	LOR	64-12	Yes
37	80	Female	OA	Charnley	11	2C	LOR	56-6	Yes
38	81	Female	OA	RM	10	3B	LOR	60-6	Yes
39	78	Male	OA	Müller	20	2C	LOR	60-6	Yes
40	64	Female	OA	PCA	12	3A	LOR	56-6	Yes
41	80	Male	OA	Charnley	18	2C	LOR	52-6	Yes
42	91	Female	OA	Plasmacup	5	2B	LOR	52-6	No
43	30	Male	OA	Profile	6	2C	LOR	56-12	Yes
44	76	Female	OA	Müller	20	3A	LOR	60-6	Yes
45	48	Male	OA	PCA	13	3A	LOR	60-6	Yes
46	53	Male	OA	PCA	17	2B	LOR	60-6	Yes

*OA* primary osteoarthritis

Charnley (Johnson & Johnson, De Puy, Warsaw, IN, USA)

Alloclassic (Centerpulse–Zimmer, Winterthur, Switzerland)

PCA (Howmedica, Rutheford, NJ, USA)

RM (Protek, Bern, Switzerland)

Profile (Johnson & Johnson)

Müller (Protek)

Ominfit (Stryker, Osteonics, Allendale NJ, USA)

Plasmacup (Aesculap, Tuttlingen, Germany)

Balgrist (Centerpulse–Zimmer)

Elite (Johnson & Johnson)

Twenty-six patients underwent acetabular revision surgery, receiving a BOFOR cup (Smith and Nephew, Plus Orthopaedics AG, Rotkreuz, Switzerland) made of titanium with a corundum-blasted surface. This is a multi-hole cup for screw placement, has cranial and caudal ribs, and the oval shape is longer over the longitudinal axis than across the anteroposterior diameter. A LOR cup (Zimmer, Sulzer Medica, Winterthur, Switzerland), a well-known device [9, 22], was implanted in nineteen patients (one bilaterally). The sizes of the implants are also shown in Table 1.

A direct lateral approach was used in 29 patients, an extended trochanteric osteotomy in 11, and a posterolateral approach in 5. The previous component, cement, and membrane were removed and the acetabular defect was confirmed intraoperatively. Different samples were sent for microbiological and histological analyses; there were no signs of acute inflammation nor postive cultures for any microorganism in any hip. Acetabular reaming was performed with hemispherical reamers until anteroposterior stability was achieved. An oblong trial component was used in order to determine the superior defect. Morselized bone allograft was used in all but five hips to fill cavitary defects, and the acetabulum was reamed reversely before implantation of the cup. Fresh-frozen femoral head allograft from the bone bank was morselized with a bone mill (Lere Bone Mill, Johnson and Johnson). Additional screws were implanted in all patients. The median number of screws was 3 (range 2–5) for both designs. All the screws were used according to the primary stability of the cup: two screws were employed if the cup was fixed after pulling out the mallet of the implant; more than two were used if there was any movement. In all cases the screws were positioned inside the iliac, but one screw was place inside the pubis in cases with a type 3A or 3B bone defect. The femoral component was revised during the same surgery in ten patients.

Intravenous antibiotic prophylaxis with cefazolin 2 g, or vancomycin 1 g in allergic patients, was administered intraoperatively and continued for three days. All patients received low-molecular-weight heparin subcutaneously for six weeks. Postoperative weight-bearing differed between patients as a function of their acetabular bone loss and associated femoral revision surgery during the same surgery. Surgeries ranged from single acetabular revisions with minimal bone defect, in which weight-bearing was initiated during the second postoperative day with two crutches as tolerated, to more complex surgeries with associated femoral revision and major bone loss, after which weight-bearing was delayed for three months.

Clinical results assessed pain, function, and range of motion according to the Merle D’Aubigné and Postel scale [28]. Standard anteroposterior radiographs of the pelvis and lateral radiographs of the hip were made immediately after the operation, at 6 weeks, at 3, 6, and 12 months, and annually thereafter, following the same protocol. The patient was positioned supine, with his/her feet together. The X-ray tube was positioned over the symphysis pubis, one meter from and perpendicular to the table, with a symmetric obturator foramen and a visible lesser trochanter and iliac crest [26]. To reduce interobserver error, measurements were performed by a single author (EGR) who had not been involved in the surgery. The cup position was evaluated by assessing the acetabular abduction angle, the height of the center of the hip (as measured from the center of the femoral head to the interteardrop line), and the horizontal distance of the cup (as measured from the center of the femoral head to the Köhler line) [19]. Acetabular reconstruction was evaluated according to the Ranawat triangle [32]. The true acetabulum region was the area enclosed by a right-angle triangle with a height and width equal to 20 % of the height of the pelvis on the AP radiograph. The midpoint of the hypotenuse coincides with the approximate center of the femoral head (AFHC) and is the center of rotation of the hip. The AFHC was used as the reference point to measure the distance to the center of the prosthetic femoral head. This distance was recorded in the preoperative and postoperative radiographs in order to assess the actual reconstruction achieved. Whether or not the center of the prosthetic femoral head was outside the triangle before and then after revision surgery was also recorded. The distribution of any radiolucent or radiodense lines or osteolysis at the acetabular bone–prosthesis interface was recorded in the three zones described by DeLee and Charnley [11]. The current method for determining radiographic aseptic loosening of the acetabular component is the appearance of radiolucent lines around the three described zones and migration of the cup, as determined by a change of >5° in the acetabular abduction angle and >3 mm in the height of the cup or in the horizontal distance [27]. All of the measurements were corrected for magnification using the known dimensions of the femoral head.

### Statistics

Statistical analysis was performed using SAS 9.1 (SAS Institute Inc., Cary, NC, USA). Quantitative data are described as mean (range). Time to re-revision and loosening was estimated by Kaplan–Meier analysis, including the 95 % confidence interval (CI) [20]; the survival curves were compared using the log-rank test. The univariate Cox model was adjusted for quantitative variables to identify the risk factors for loosening. The level of significance was *p* < 0.05.

## Results

### Complications

There were two early dislocations in the postoperative period that were treated successfully with conservative treatment. There were also two superficial infections that were controlled with antibiotics and local management. There were two intraoperative femoral fractures that were solved with cerclage and a long femoral stem. All of these hips were included in the study.

### Clinical results

There were four re-revisions: one LOR cup due to recurrent dislocation (case 46), who was revised to a constrained liner, and three cases (25, 29, and 31) due to aseptic loosening. Case 25 was an 80-year-old female patient with a loosened Ominift (Stryker, Osteonics, Allendale, NJ, USA) uncemented cup and a type 2C bone defect who was revised to a BOFOR cup. She had referred pain during the first postoperative year, with an aseptic loosened cup on the radiographs, so she was re-revised two years later. Case 29 was a 74-year-old female patient with a loosened Balgrist (Centerpulse, Winterthur, Switzerland) cup who had a type 3A bone defect and was revised to a LOR oblong cup that became loose during the second postoperative year. Case 31 was a 76-year-old female patient with a loosened PCA (Howmedica, Rutherford, NJ, USA) cup and a type 3A bone defect who had received a LOR oblong cup that became loose during the sixth postoperative year. In each of these cases, the re-revision surgery employed impaction bone grafting and a cemented cup was placed at the time of loosening in each case.

**Fig. 1:**
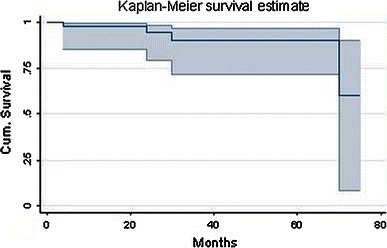
Graph showing the Kaplan–Meier cumulative probability that re-revision surgery of the cup for the implants was not included in the follow-up study. The *upper* and *lower curves* represent the 95 % confidence intervals

The survival rate for re-revision of the cup for aseptic loosening at 75 months was 60.1 % (95 % CI 11.45–100) (Fig. 1). With the number of hips available, no significant differences were found between the two types of implanted cup (*p* = 0.1993). Clinical results improved by 5.2–15.2 points according to the Merle D’Aubigné and Postel scale, although six patients related groin pain during daily activities such as putting on shoes or standing from sitting in a chair.

### Radiological analysis

**Fig. 2a–b:**
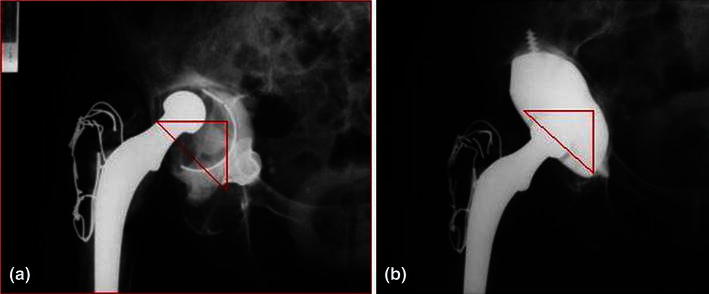
Radiographs showing acetabular reconstruction using a BOFOR cup. **a** Preoperative radiograph showing the center of rotation outside Ranawat’s triangle. **b** Postoperative radiograph showing the center of rotation inside Ranawat’s triangle

Regarding the acetabular reconstruction, Table 2 shows all of the radiological parameters evaluated for each patient. The radiological analysis showed a mean preoperative acetabular abduction angle of 65.9° (range 35–100), a mean horizontal distance of 34.7 mm (range 5–60), and a mean vertical distance to the center of the femoral head of 34.3 mm (range 5–70). After hip revision surgery, the mean postoperative acetabular abduction angle was 48.6° (range 35–80), the horizontal distance was 31.5 mm (range 5–40), and the height of the center of the hip was 23.2 mm (range 5–45). We also observed that, on the preoperative radiographs, 33 hips were outside Ranawat’s triangle and 12 were inside, while on the postoperative radiographs 37 were inside and 8 were outside (Fig. 2). The mean distance between the center of the femoral head prosthesis and the AFHC improved from 21.5 mm (range 5–45) to 10.2 mm (range 0–25) (Table 3). Acetabular reconstruction was achieved in most hips regardless of bone defect (Table 4). The mean height of the center of the hip showed greater improvement with bone defect types 2C and 3 than with type 2B; the other parameters—the acetabular abduction angle, the horizontal distance, and the mean CPFH–AFHC distance—improved in the same manner.

**Table 2:** Preoperative and postoperative radiological data at the last follow-up evaluation

Case no.	Preoperative acetabular abduction angle (°)	Preoperative horizontal distance (mm)	Preoperative vertical distance (mm)	Preoperative CPFH–AFHC distance (mm)	True acetabular region (preoperative)	Postoperative acetabular abduction angle (°)	Postoperative horizontal distance (mm)	Postoperative vertical distance (mm)	Postoperative CPFH–AFHC distance (mm)	True acetabular region (postoperative)
1	75	30	25	12	Inside	45	35	20	8	Inside
2	38	40	25	15	Outside	60	35	20	10	Outside
3	55	40	20	15	Outside	50	30	20	10	Inside
4	110	22	55	30	Outside	70	30	15	3	Inside
5	85	30	35	20	Outside	55	35	5	20	Inside
6	70	30	40	20	Outside	50	30	15	13	Outside
7	45	30	20	8	Inside	35	32	22	10	Inside
8	45	40	20	18	Outside	35	35	20	8	Inside
9	35	15	35	10	Outside	40	15	35	15	Inside
10	70	30	15	7	Outside	45	35	0	25	Outside
11	100	30	45	25	Outside	40	30	20	4	Inside
12	60	30	10	12	Outside	70	30	10	3	Inside
13	95	30	50	35	Outside	80	35	35	20	Inside
14	70	35	30	10	Inside	45	35	10	15	Outside
15	45	35	20	10	Inside	50	35	20	2	Inside
16	50	40	40	25	Outside	50	35	20	5	Inside
17	80	35	30	10	Inside	40	35	15	5	Inside
18	55	30	20	12	Inside	50	35	20	10	Inside
19	50	40	35	20	Outside	50	40	25	5	Outside
20	70	30	10	15	Outside	50	35	5	10	Outside
21	40	35	35	5	Inside	45	30	20	3	Inside
22	55	30	25	5	Inside	40	30	20	2	Inside
23	65	30	35	20	Outside	50	35	20	5	Inside
24	70	30	40	20	Outside	40	25	25	0	Inside
25	80	35	25	10	Inside	45	30	20	2	Inside
26	35	30	55	37	Outside	50	30	40	15	Inside
27	80	60	45	40	Outside	55	35	40	20	Inside
28	50	40	20	20	Outside	45	30	45	25	Outside
29	70	35	50	30	Outside	70	5	5	25	Outside
30	70	35	30	10	Outside	40	30	20	0	Inside
31	70	40	40	20	Inside	70	35	40	20	Inside
32	50	40	40	30	Outside	60	30	25	4	Inside
33	55	42	36	26	Outside	46	30	30	8	Inside
34	80	40	40	25	Outside	45	30	20	5	Inside
35	55	40	45	30	Outside	45	30	35	13	Inside
36	80	40	70	45	Outside	40	30	35	20	Inside
37	35	35	50	35	Outside	45	35	45	25	Outside
38	80	35	45	30	Outside	40	25	35	15	Inside
39	80	40	40	30	Outside	50	40	20	15	Inside
40	80	35	25	30	Outside	45	25	20	5	Inside
41	68	34	24	18	Outside	42	26	34	18	Inside
42	55	38	28	32	Outside	45	30	30	3	Inside
43	80	35	40	20	Inside	45	20	35	10	Inside
44	40	35	35	28	Outside	50	30	30	12	Inside
45	55	45	25	30	Inside	42	40	15	6	Inside
46	62	34	18	6	Inside	49	33	15	5	Inside

*CPFH* center of the prosthetic femoral head, *AFHC* approximate center of the femoral head

**Table 3:** Preoperative and postoperative prosthetic femoral head locations

	Preoperative	Postoperative	*p* value
Mean acetabular abduction angle (°)	65.9 (35–100)	48.6 (35–80)	<0.001
Mean horizontal distance (mm)	34.7 (5–60)	31.5 (5–40)	0.004
Mean vertical distance (mm)	34.3 (5–70)	23.2 (5–45)	<0.001
Mean CPFH–AFHC distance (mm)	21.5 (5–45)	10.2 (0–25)	<0.001
Inside Ranawat’s triangle (number of hips)	12	37	
Outside Ranawat’s triangle (number of hips)	33	8	

*CPFH* center of the prosthetic femoral head, *AFHC* approximate center of the femoral head

**Table 4:** Preoperative and postoperative prosthetic femoral head locations as a function of bone defect type

Bone defect type [13]	2B *n* = 17	2C *n* = 12	3 *n* = 17	*p* values
Acetabular abduction angle (mean ± SD)				
Preoperative	54.1 ± 13.7	69.5 ± 16.1	72.3 ± 19.2	<0.001
Postoperative	45.9 ± 6.5	50.8 ± 11.04	52.3 ± 12.3	0.259
Horizontal distance (mean ± SD, in mm)				
Preoperative	35.7 ± 4.7	37.5 ± 8.1	31.6 ± 9.6	0.004
Postoperative	31.9 ± 4.4	33.7 ± 3.7	29.3 ± 9.4	0.78
Vertical distance (mean ± SD, in mm)				
Preoperative	24.8 ± 8.3	37.5 ± 10.5	39.3 ± 16.4	<0.001
Postoperative	21.2 ± 9.5	25.4 ± 12.8	22.3 ± 12.2	0.006
CPFH–AFHC distance (mean ± SD, in mm)				
Preoperative	16.8 ± 9.2	23.1 ± 11.6	24.5 ± 9.1	<0.001
Postoperative	7.3 ± 6.6	13.5 ± 8.8	10.7 ± 7.4	0.46

**Fig. 3a–b:**
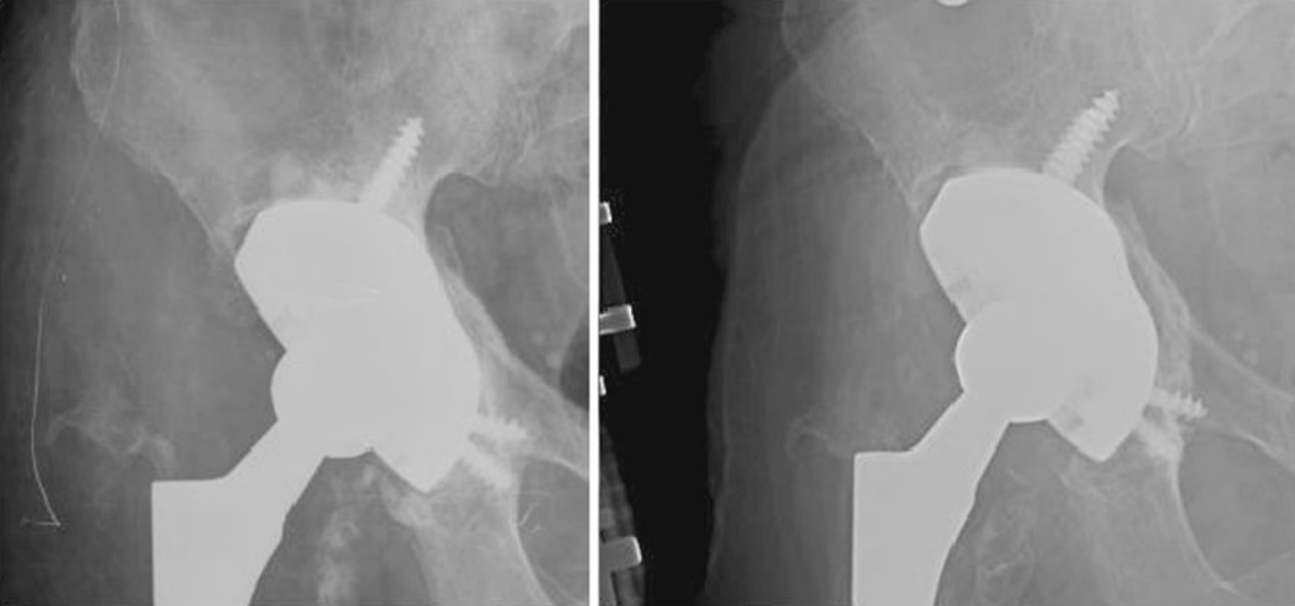
Radiographs of a 75-year-old-man, case 23. **a** Postoperative radiograph after acetabular reconstruction using a BOFOR cup. **b** Appearance of a complete radiolucent line and a slight change in the position of the cup at four years (the patient used a cane and related mild groin pain)

**Fig. 4:**
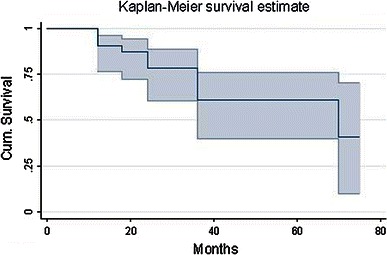
Graph showing the Kaplan–Meier cumulative probability that radiological cup loosening was not seen for the implants included in the follow-up study. The *upper* and *lower curves* represent the 95 % confidence intervals

Thirteen cups showed radiological loosening (Fig. 3). The survival rate for radiological loosening at 75 months was 40.54 % (95 % CI 15.8–75.21) (Fig. 4). We did not observe any differences between the two assessed designs (*p* = 0.48). Radiolucent lines were recorded in seven hips—all in zones 2 and 3. No radiodense lines or osteolysis were observed in this series.

With the hips available for analysis, bone defect was not related to aseptic radiological cup loosening: the survival rate for aseptic loosening at seven years in hips with a type 2B bone defect was 59.1 % (95 % CI 31–89), 84.6 % (CI 95 % 69–100) with type 2C, and 49.1 % (CI 95 % 20–80) with type 3 (*p* = 0.63). Regarding postoperative acetabular reconstruction, according to the univariate Cox model, a smaller horizontal distance was correlated with the appearance of radiological cup loosening (*p* = 0.017; hazard ratio, 95 % CI 87.1–98.6). The other radiological parameters for cup position, such as acetabular abduction angle and vertical distance, were not related to radiological cup loosening. We also observed that the mean postoperative distance between the center of the femoral head prosthesis and the AFHC was 10.1 mm for fixed cups and 12.1 mm for loosened cups (*p* = 0.68); also, among the eight cups that were outside the Ranawat triangle, three became loose (*p* = 0.51). Data on loosened cups are shown in Table 5.

**Table 5:** Loosened cups

Case no.	Time until radiological loosening (months)	Oblong cup	Bone defect	Revision surgery	Postoperative acetabular abduction angle (°)	Postoperative horizontal distance (mm)	Postoperative vertical distance (mm)	Postoperative CPFH–AFHC distance (mm)	True acetabular region (postoperative)
3	12	BOFOR	3A	No	50	30	20	10	Inside
12	24	BOFOR	3A	No	70	30	10	3	Inside
13	12	BOFOR	2C	No	80	35	35	20	Inside
14	36	BOFOR	2C	No	45	35	10	15	Outside
15	24	BOFOR	2B	No	50	35	20	2	Inside
22	36	BOFOR	2B	No	40	30	20	2	Inside
23	36	BOFOR	3A	No	50	35	20	5	Inside
24	24	BOFOR	2B	No	40	25	25	0	Inside
25	12	BOFOR	2C	Yes	45	30	20	2	Inside
29	18	LOR	3A	Yes	70	5	5	25	Outside
30	12	LOR	2B	No	40	30	20	0	Inside
31	72	LOR	3A	Yes	70	35	40	20	Inside
38	36	LOR	3B	No	40	25	35	15	Inside

## Discussion

It is well known that cup loosening produces cup migration and bone loss in both cemented [14] and uncemented [6, 16] THA. Different options are being used to manage acetabular bone defects: techniques such as impaction bone grafting and a cemented cup (according to Slooff et al.) provide excellent results [13, 33], reinforcement rings have also been used with different results in large defects [3, 7, 38], as have trabecular metal cups and augments [24]. Although uncemented hemispherical cups are a valid option in small acetabular defects, it is accepted that they provide poor results when acetabular bone defects are >50 % [12]. Although extra-large uncemented components have achieved good results, the extensive reaming required in order to obtain good bone contact with the host bone (which is more important in the anteroposterior diameter of the acetabulum) can ultimately affect implant stability [39]. The purpose of an oblong cup is to obtain enough stability in both the anterior and posterior column of the acetabulum without sacrificing the longitudinal axis [9, 22]. Since achieving an anatomic center of hip rotation is desirable in order to obtain good results in acetabular revision surgery, we assessed the clinical and radiological results of two different types of oblong cup with regard to the preoperative bone loss and postoperative radiological position of the cup after surgery.

Different authors have reported good clinical and radiological results using the LOR cup [22, 36]. Herrera et al. [18] reported a 14.2 % migration rate that was greater in vertical cups and in major bone defects with incomplete cup contact at the acetabular rim; all cases were combined defects or presented a pelvic discontinuity. Landor et al. [23] reported a survival rate for aseptic loosening of 90 % at 12 years without deep infection cases in patients with different bone defects. The survival rate for cup loosening here is not better than the reports mentioned above. We also observed that there were no differences between the two devices evaluated. As far as we know, there are no articles regarding outcome with BOFOR cups. Other types of implants—such as the bilobed cups used in hip revision surgery—provide different results, although the number of cases is not very large [1, 5, 29]. Although bilobed acetabular revision components are different, they also try to fill overlying defects and relocate the hip rotation center. Chen et al. [5] reported an early rate of probable or definite loosening of 24 % in a follow-up that ranged from 24 to 41 months; failure was greater with major bone defects and undersized components, and they did not recommend the routine use of these types of implants. Abeyta et al. reported the long-term results of 15 hips using S-ROM (DePuy Johnson & Johnson, Leeds, UK) oblong bihemispherical cups; three cups were revised due to aseptic loosening, and one showed complete radiolucency around the cup [1]. On the other hand, Moskal et al. [29] assessed 11 bilobed components in combined acetabular defects that did not require revision over a five-year follow-up. Although most of series have shown good results for oblong or bilobed cups, Babis et al. [2] recently observed poor results for the PROCOTYL E cup (Wright Medical Technology, Arlington, TN, USA) in Paprosky defect type 3A, and they do not recommend this technique (Table 6). In our study, we also observed that most patients with radiological cup loosening reported mild groin pain, and some needed a cane or two crutches, but they usually refused re-revision surgery due to their age and low activity level. This is a frequent observation, since migration is slow and clinical consequences are not severe, so a period of observation is possible [14].

**Table 6:** Clinical studies of oblong cups in revision hip surgery

References	Number of hips	Type of cup	Preoperative bone defect [31]	Mean follow-up (years)	Aseptic loosening (%)
Surace et al. [36]	41	LOR	2A–3B	5.1	0
Herrera et al. [18]	35	LOR	III, IV^a^	6.3	14.2
Landor et al. [23]	133	LOR	2B–3B	9.7	8.3
Chen et al. [5]	37	Bilobed cup	2A–3B	3.5	24
Moskal et al. [29]	11	S-ROM	III^a^	6	0
Abeyta et al. [1]	15	S-ROM	III^a^	11	20
Babis et al. [2]	62	Procotyl E	3A	5	35
Current study	46	26 BOFOR 20 LOR	2B–3B	7.2	28

^a^AAOS classification

We also evaluated the reconstruction of the center of rotation of the hip achieved with these devices. Both an anatomic hip center and maximum bone host contact are desirable postoperatively to support stability. Some authors have reported excellent long-term results using the high hip center technique [4, 17, 34], but other series have reported a higher aseptic loosening rate for a nonanatomical acetabular component site [30, 39]. Finite-element analysis of a protruded acetabulum has shown that stress on the deficient medial wall varies as a directly function of medial placement of the cup [8]. Different authors report that correcting a deficient acetabulum to the anatomical position is crucial for achieving good long-term results [21, 30, 39]. In our study, a good acetabular reconstruction was obtained in most cases. Although the number and positions of the screws were chosen according to the primary stability of the cup, a screw fixed to the pubis using other devices can be recommended, since we only used this in bone defects of type 3A or 3B, and this could have influenced the results [7]. After the surgery, most of the hips were inside the Ranawat triangle, and the postoperative distance to the AFHC improved.

Despite these findings, a high rate of radiological loosening was observed. Many factors may be responsible for acetabular cup loosening. Surace et al. [36] found that the clinical results of the LOR cup were correlated with whether the postoperative position was correct. Theoretically, the location of the center of rotation of the hip affects the load, and a higher and more medial position will result in a greater load than a lower placement [39]; in our series, we also observed a relationship between a more medial cup and the appearance of loosening, so there may be a technical issue. The use of a morselized bone allograft did not improve the rate of loosening either, but the technique used is different from impaction bone grafting [13, 33], and in our series the chip sizes were smaller, and they were only used to fill cavitary defects. Regarding bone defect, several authors have reported worse results in major bone defects using oblong cups [18, 22, 23]. We did not use these implants when there was a pelvic discontinuity. We realize that minor bone defects could have been treated with an hemispherical cup; however, the surgeons considered using these oblong cups when there was a superior defect on the acetabular rim before or after the old implant was removed. With the number of hips available in our study, we did not observe any difference between the types of bone defect; thus, the rate of radiological cup loosening with oblong cups was high regardless of whether there was a minor or a major bone defect.

The most important limitation of our study is the small number of patients. This may be one of the reasons that some of the variables assessed, such as intraoperative bone defect and some of the data regarding the postoperative position of the cup, did not influence the results. This study is retrospective and could not have been randomized. There is a lack of some clinical data, such as leg length discrepancy or limp. In order to simplify and focus our analysis, given that the central hypothesis of our study was the stable bone fixation of these implants, we only used the Merle D’Aubigné and Postel scale, as mentioned above. Being able to detect prosthetic movement at an early stage and over time could be important for predicting later cup loosening in cases using uncemented cups in revision THA. RSA analysis could detect migration and rotation of the cup at an early stage and over time [25]. However, we have only used conventional radiographs, and we realize the limitations of these measurements. Another limitation of this study is the lack of a control group of patients with similar ages and acetabular defects who were operated on with other techniques.

In our series, the clinical and radiological results for these oblong cup designs were not encouraging at a medium-term follow-up. We observed a high rate of radiological loosening, and this is a concern given that this failure was observed regardless of the grade of the bone defect. Although a reconstruction of the center of rotation of the hip was frequently achieved, and the postoperative position was frequently correct, this was not enough to obtain a good rate of radiological loosening with these cups. A larger postoperative horizontal distance may have improved the results. Since these oblongs cups are still available for many orthopedic surgeons, we recommend careful evaluation of the patient before these types of devices are used for revision hip surgery. We currently recommend using other validated techniques, such as a hemispherical uncemented cup for minor defects or bone impaction grafting and a cemented cup for larger defects. Only long-term results can provide sufficient data to reach definite conclusions.
